# Pharmacological prevention and early treatment of post-traumatic stress disorder and acute stress disorder: a systematic review and meta-analysis

**DOI:** 10.1038/s41398-019-0673-5

**Published:** 2019-12-09

**Authors:** Laurence Astill Wright, Marit Sijbrandij, Rob Sinnerton, Catrin Lewis, Neil P. Roberts, Jonathan I. Bisson

**Affiliations:** 1 Division of Psychological Medicine and Clinical Neurosciences, Cardiff University School of Medicine, Cardiff, UK; 2 Department of Clinical Psychology, VU University Amsterdam, Amsterdam, Netherlands; 3 Directorate of Psychology and Psychological Therapies, Cardiff & Vale University Health Board, Cardiff, UK

**Keywords:** Pathogenesis, Psychiatric disorders, Clinical pharmacology

## Abstract

Post-traumatic stress disorder (PTSD) is a common mental disorder associated with significant distress and reduced functioning. Its occurrence after a severe traumatic event and association with characteristic neurobiological changes make PTSD a good candidate for pharmacological prevention and early treatment. The primary aim for this systematic review and meta-analysis was to assess whether pharmacological interventions when compared to placebo, or other pharmacological/psychosocial interventions resulted in a clinically significant reduction or prevention of symptoms, improved functioning or quality of life, presence of disorder, or adverse effects. A systematic search was undertaken to identify RCTs, which used early pharmacotherapy (within three months of a traumatic event) to prevent and treat PTSD and acute stress disorder (ASD) in children and adults. Using Cochrane Collaboration methodology, RCTs were identified and rated for risk of bias. Available data was pooled to calculate risk ratios (RR) for PTSD prevalence and standardised mean differences (SMD) for PTSD severity. 19 RCTs met the inclusion criteria; 16 studies with adult participants and three with children. The methodological quality of most trials was low. Only hydrocortisone in adults was found to be superior to placebo (3 studies, *n* = 88, RR: 0.21 (CI 0.05 to 0.89)) although this was in populations with severe physical illness, raising concerns about generalisability. No significant effects were found for the other pharmacotherapies investigated (propranolol, oxytocin, gabapentin, fish oil (1470 mg DHA/147 mg EPA), fish oil (224 mg DHA/22.4 mg EPA), dexamethasone, escitalopram, imipramine and chloral hydrate). Hydrocortisone shows the most promise, of pharmacotherapies subjected to RCTs, as an emerging intervention in the prevention of PTSD within three months after trauma and should be a target for further investigation. The limited evidence for hydrocortisone and its adverse effects mean it cannot be recommended for routine use, but, it could be considered as a preventative intervention for people with severe physical illness or injury, shortly after a traumatic event, as long as there are no contraindications. More research is needed using larger, high quality RCTs to establish the most efficacious use of hydrocortisone in different populations and optimal dosing, dosing window and route. There is currently a lack of evidence to suggest that other pharmacological agents are likely to be effective.

## Introduction

Post-traumatic stress disorder (PTSD) is a common mental disorder manifesting through symptoms of re-experiencing, hyper-arousal and avoidance following a traumatic event. In high-risk populations the prevalence of PTSD is estimated at 15.4%^1^. PTSD is associated with substantial physical and psychiatric co-morbidity, including substance abuse and suicide^2^. The DSM-5 states that symptoms must be present for one month following the traumatic event for PTSD to be diagnosed^3^. Acute stress disorder (ASD) has similar symptoms to PTSD, is diagnosed 3 days to 1 month post-trauma and is a good predictor of PTSD^4^. Therapies to prevent early traumatic stress reactions developing into chronic PTSD, particularly in high-risk individuals, are needed to alleviate this significant morbidity.

While some psychological interventions to prevent the development of PTSD are ineffective^5^ and others, such as psychological debriefing after trauma may even be harmful^6^, there is evidence of benefit of trauma-focused cognitive behavioural therapy in treating individuals with acute traumatic stress symptoms^7^ and preliminary work on prolonged exposure therapy in the immediate aftermath of trauma, has shown promise in the reduction of post-traumatic stress reactions^8^. The limited evidence available for treatments incorporating both psychological and pharmacological intervention, however, has so far failed to show significant benefit^9^.

As our scientific understanding of the neurobiological changes occurring during PTSD onset has increased, more research has focused on pharmacological interventions to prevent PTSD. For example, the finding that memory consolidation appears particularly vulnerable to disruption in the six hours after trauma^10^, makes the shifts in neurobiological activity in these “golden hours”^11^ and beyond a promising target for pharmacological intervention^12^.

Early research explored the effects of benzodiazepine administration and was largely ineffective^13^. Later research has focused on B-blockers, such as propranolol, and their ability to disrupt post-synaptic norepinephrine receptors^14^. Studies have suggested that human participants who received propranolol have decreased recall of emotionally stimulating material, possibly due to the blocking of memory consolidation^15^ and subsequent studies have explored the efficacy of propranolol as a preventive agent^16^.

Other studies have found an association between low cortisol levels following motor vehicle accidents and subsequent PTSD^17,18^. Both human and animal studies suggest that glucocorticoids attenuate heightened fear response^19^ through increased removal of fear inducing memories^20^ resulting in interest in the potential preventive effects of hydrocortisone.

In addition to propranolol and hydrocortisone, observational studies have associated early morphine use with reduced rates of PTSD^21^ and other drugs, including SSRIs^22^, gabapentin^23^, α-omega fatty acids^24^ and ketamine^25^ have also been investigated for the prevention of PTSD. Previous systematic reviews in this area have highlighted both the sparsity and the poor quality of the trials^24,26^. Sijbrandij et al.^26^ included randomised controlled trials (RCTs), controlled clinical trials and cohort studies to maximise the number of included studies. After the meta-analyses of Sijbrandij et al.^26^ and Amos et al.^24^ more recent studies have been published, such as a study on escitalopram^22^ and a study on oxytocin^27^. In order to determine if the evidence has developed in the last few years, we undertook a further systematic review and meta-analysis, building on the original review of Sijbrandij et al.^26^ but only including RCTs. The inclusion of just RCTs allowed us to focus on a higher quality of evidence to offer sound recommendations for future research. Another difference is that we included studies which administered the intervention within the first three months instead of just one month of the trauma. The study was conducted to update the International Society of Traumatic Stress Studies 2018 Treatment Guidelines^28^, the scoping question for which investigated early pharmacological intervention within three months of the traumatic event.

Thus the primary aim for this systematic review and meta-analysis was to assess whether pharmacological interventions when compared to placebo, or other pharmacological/psychosocial interventions resulted in a clinically significant reduction or prevention of symptoms, improved functioning or quality of life, presence of disorder, or adverse effects. We sought to analyse different classes of pharmacological agents separately, rather than pooling the data.

## Methods

We adopted a methodology based on the Cochrane Handbook for Systematic Reviews of Interventions^29^ and a PRISMA checklist was completed.

### Selection criteria

The inclusion criteria were: (a) any RCT (including cluster and cross-over trials); (b) investigating the effects of pharmacological intervention delivered within three months of the traumatic event; (c) when compared to placebo, pharmacological or psychosocial interventions; (d) in participants exposed to a traumatic event likely to meet the A criterion for DSM5 PTSD; and (e) PTSD or ASD symptoms measured using one or more validated clinician administered or self-report outcome measures. There was no restriction on the severity of PTSD/ASD symptoms or the type of traumatic event, no restriction on sample size, and both published and unpublished studies were eligible for inclusion. Only studies published in English were included.

### Search strategy and selection criteria

For this review we combined an updated search undertaken by the Cochrane Collaboration with the original search strategy from the review of Sijbrandij et al.^26^. This systematic review was undertaken alongside a number of other reviews carried out to update the ISTSS Treatment Guidelines^28^. As part of this all RCTs related to the prevention and treatment of PTSD from 2013 to the 31st May 2018 were identified and scrutinised, producing 16 new papers on early pharmacotherapy considered in more detail here. For this updated search the inclusion criteria were reconsidered and subsequently much broader. The time frame for intervention was extended to 3 months post-trauma. The original search strategy used PubMed, PsycINFO, Embase and the Cochrane database of randomised trials with no limitation on start date. Terms referring to PTSD were combined with terms referring to pharmacotherapy (using both MeSH terms and text words) and we also checked the references of four narrative reviews of pharmacological prevention of PTSD^5,12,16,30^. Details of the searches and exact search strings are provided in the appendix.

All titles and abstracts were appraised by two independent screeners and any disagreements were discussed. The full text of any potentially relevant papers was acquired and if we were unable to locate the full text for any study the corresponding author was contacted to request the paper. To determine if potentially relevant studies met the inclusion criteria the full text was independently reviewed by two authors (L.A.W. and J.I.B.).

### Data extraction

Data were extracted by two independent reviewers (L.A.W. and J.I.B.) using identical data extraction forms. There were only minor irregularities between reviewers, which were discussed and consensus agreed on. Study authors were contacted if more information was required. Basic demographic data and details of the intervention used was collected along with primary outcomes of PTSD/ASD symptom severity and incidence.

### Assessment of study bias

The Cochrane Collaboration’s tool for assessing risk of bias in randomised trials’^31^ was used for each identified study. This tool assesses the likelihood of bias in randomised trials, including the adequate generation of allocation sequence, acceptable concealment of allocation, satisfactory blinding of participants and personnel, and assessing the degree of incomplete outcome data. Risk of bias was assessed by two independent reviewers (L.A.W. and J.I.B.) and any disagreement resolved by discussion. These ratings were considered and a GRADE judgement (which assesses quality of evidence to make recommendations for clinical practice^32^) was presented for each outcome.

### Synthesis of results

The primary outcome in the meta-analysis was reduction in PTSD symptoms 3–6 months after the traumatic event (but it was agreed a priori that the nearest time point to this would be accepted, and if there was no measure of PTSD symptoms, ASD symptoms would be included instead). This was performed using a random-effects model. PTSD incidence at 3–6 months after the traumatic event was also considered. For PTSD/ASD incidence we calculated risk ratios whereas for severity we calculated standardised mean differences, along with associated confidence intervals. For outcomes including more than one study we measured statistical heterogeneity by calculating the I^2^ statistic (*t*). As this was low for all results it did not change our analyses. Data was pooled if outcomes included two or more studies. Sub-group analyses were not performed as there were few studies for individual outcomes. All analyses were done using the Cochrane Collaboration’s Review Manager 5.3 software^33^.

## Results

**Fig. 1:**
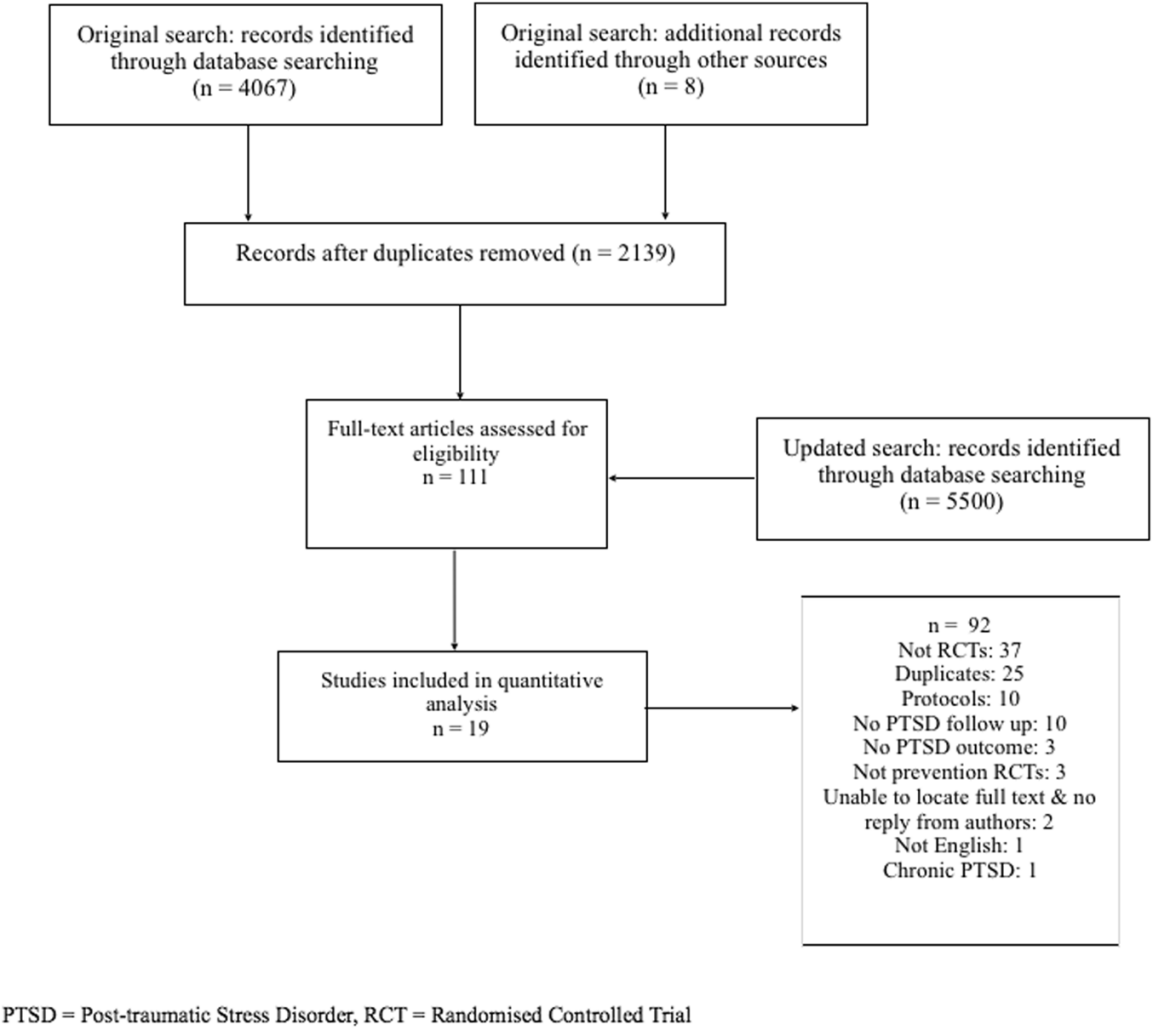
PRISMA flow diagram.

The initial search produced 2139 papers. The updated search was broader and contained 5500 additional papers. We examined the full text of 111 papers and 18 of these met the inclusion criteria. One other text which met the inclusion criteria was highlighted during peer review^34^. The other 93 were excluded as per Fig. 1.

Our systematic review identified 19 RCTs with a total of 3629 participants. There were 16 adult RCTs^16,20,23,27,34–45^ (*n* = 3387) and three child RCTs^46–48^ (*n* = 242). 16 trials were included in the meta-analysis, with three excluded^39,41,44^ as they lacked sufficient information to include. For example, Schelling et al.^41^ only included the median scores (with IQR) for PTSD severity. Tables 1 and 2 show detailed characteristics of these trials. All studies focused on early intervention to prevent PTSD/ASD apart from Shalev et al.^36^ and Suliman et al.^22^ which were early treatment trials. Interventions were classified as early treatment trials if the intervention extended beyond the first month post-trauma. Shalev et al.^42^ initiated treatment with only those participants who met DSM-IV PTSD diagnostic criteria (assessed at a mean of 19.8 days post-trauma). Similarly, Suliman et al.^22^ included participants meeting the DSM IV criteria for full or partial ASD, initiating treatment within 28 days. When measuring outcomes from all trials, a clinician administered measure was used where available and self-report questionnaires if not^36,41,43^.

**Table 1:** Characteristics of included adult studies.

	Country	Trauma sample	Pharmacotherapy	Timing after trauma	Comparator	*n*	PTSD/ASD outcome	Follow up
Delahanty et al.*	USA	Injury	Hydrocortisone	<12 h	Placebo	43	PTSD Severity (CAPS)	1, 3 months
Denke et al.*	Germany	Septic Shock	Hydrocortisone	<6 h	Placebo	18	PTSD Incidence (PTSS-10)	12 months
Schelling et al.*	Germany	Septic Shock	Hydrocortisone	<6 h	Placebo	20	PTSD Incidence & Severity (SCID-IV & PTSS-10)	31 months
Schelling et al.	Germany	Zohar et al.*	Hydrocortisone	<6 h	Standard therapy	48	PTSD Severity (PTSS-10)	6 months
Weis et al.*	Germany	Cardiac Surgery	Hydrocortisone	<6 h	Placebo	28	PTSD Incidence (PTSS-10)	6 months
Zohar et al.*	Israel	Injury	Hydrocortisone	<6 h	Placebo	17	PTSD Incidence (CAPS)	3 months
Kok et al.*	Netherlands	Cardiac Surgery	Dexamethasone	<6 h	Placebo	2458	PTSD Incidence (PTSS-10)	18–48 months
Hoge et al.*	USA	Injury	Propranolol	4–12 h	Placebo	41	PTSD Incidence & Severity (CAPS)	1, 3 months
Pitman et al.*	USA	Injury	Propranolol	<6 h	Placebo	24	PTSD Incidence & Severity (CAPS)	1, 3 months
Stein et al.*	USA	Injury	Propranolol	<48 h	Placebo	38	ASD Severity & PTSD Incidence (ASDS & PCL-C)	1, 4, 8 months
Shalev et al.*	Israel	Injury	Escitalopram	19.8 days	Placebo	36	PTSD Incidence & Severity (CAPS)	5, 9 months
Suliman et al.*	South Africa	Injury	Escitalopram	<28 days	Placebo	29	PTSD Incidence (CAPS)	0.5, 6, 14 months
Zohar et al.	Israel/South Africa	Injury	Escitalopram	<30 days	Placebo	198	PTSD Severity (CAPS)	14 months
Van Zuiden et al.*	Netherlands	Injury	Oxytocin	<12 days	Placebo	107	PTSD Incidence & Severity (CAPS)	1.5, 3, 6 months
Matsuoka et al.*	Japan	Injury	Fish oil (1470 mg DHA/147 mg EPA)	10 days	Placebo	110	PTSD Incidence & Severity (Clinical Diagnosis & CAPS)	3 months
Nishi et al.	Japan	Disaster Relief	Fish oil (224 mg DHA/22.4 mg EPA)	Not specified	Psychoeducation	172	PTSD Incidence (IES-R)	12.6 weeks

*Included in meta-analysis, PTSD, post-traumatic stress disorder, ASD, acute stress disorder, *n*, number of participants included at final assessment, CAPS, clinician administered PTSD scale,

PTSS-10, post-traumatic 10 stress symptom Inventory, IES-R, impact of events scale—revised, SCID-IV, structured clinical interview for DSM IV, ASDS, acute stress disorder scale,

PCL-C, PTSD checklist—civilian version

**Table 2:** Characteristics of included child and adolescent studies.

	Country	Trauma sample	Pharmacotherapy	Timing after trauma	Comparator	*n*	PTSD/ASD outcome	Follow up
Nugent*	USA	Injury	Propranolol	<12 h	Placebo	20	PTSD Incidence & Severity (CAPS-CA)	6 weeks
Rosenberg et al.*	USA	Burns	Propranolol	<2 days	Standard therapy	197	PTSD Incidence (MAGIC)	7 years
Robert et al.*	USA	Burns	Imipramine	36 days	Chloral hydrate	25	ASD Severity (Clinical Interview)	36 days

*Included in meta-analysis, PTSD, post-traumatic stress disorder, ASD, acute stress disorder, *n*, number of participants included at final assessment,

CAPS-CA, clinician administered PTSD scale for children & adolescents, MAGIC, Missouri Assessment of Genetics Interview for Children,

PTSD Section, DICA, diagnostic interview for children & adolescents

Seventeen studies compared effectiveness on PTSD outcomes, two studies on ASD severity^23,47^. Robert et al.^47^ assessed ASD severity after administering imipramine to one group, and chloral hydrate to another, the participants were between 5 days and 148 days (mean: 36 days) post-trauma (thermal injury). Robert et al.^47^ justified their use of ASD by arguing that acute hospitalisation for burns is continuously traumatising, and that the traumatic event continued until discharge. This is, however, debatable and at odds with commonly accepted definitions; PTSD would likely have been a more appropriate primary outcome to have measured. Stein et al.^23^ compared both ASD and PTSD outcomes, finding no significant difference across the three groups—propranolol, gabapentin and placebo.

### Risk of bias assessments

The quality of the RCTs was highly variable and the majority of the studies had areas of significant risk of bias in their methodology (explored in Supplementary Table 1 and Supplementary Table 2). Only four studies^27,34,38,39^ used intention-to-treat (ITT) analysis with the other 15 studies using completer-only analysis (Suliman et al.^22^ used a modified ITT analysis, with only completer-only analysis reported and, therefore, used in this meta-analysis and so it is not considered a true ITT trial).

### Meta-analyses

The results of our meta-analyses are shown in Tables 3 and 4. Rosenberg et al.^48^ and Sharp et al.^49^ used the same data set with different follow up periods, because of this we included the Rosenberg et al. study^48^ which used PTSD outcomes. Sharp et al.^49^ used ASD outcomes and also noted no significant difference between control and treatment groups.

**Table 3:** Effects of pharmacotherapy for PTSD prevention in adult participant RCTs.

Pharmacotherapy	Outcome	Comparisons	Participants (*n*)	RR/SMD (95% CI)	*I*^2^	GRADE judgement
Hydrocortisone	PTSD 3–6 months	3	98	RR: 0.21 (0.05 to 0.89)	0%	Low
Hydrocortisone	PTSD severity 3–6 months	1	43	SMD: −0.63 (−1.25 to −0.02)	NA	Very low
Hydrocortisone	PTSD >6 months	2	38	RR: 0.44 (0.16 to 1.23)	59%	Very low
Dexamethasone	PTSD 18–48 months	1	2458	RR: 0.80 (0.56 to 1.14)	NA	Very low
Propranolol	PTSD 3–6 months	3	96	RR: 0.75 (0.31 to 1.83)	0%	Low
Propranolol	PTSD severity 3–6 months	2	52	SMD: 0.06 (−0.49 to 0.61)	0%	Low
Escitalopram (treatment not prevention)	PTSD 3–6 months	2	92	RR: 1.05 (0.61 to 1.79)	0%	Low
Escitalopram (treatment not prevention)	PTSD severity 3–6 months	2	68	SMD: −0.01 (−0.49 to 0.47)	0%	Low
Gabapentin*	PTSD 3–6 months	1	32	RR: 0.80 (0.18 to 3.59)	NA	Very low
Oxytocin*	PTSD severity 3–6 months	1	107	SMD: −0.24 (−0.62 to 0.14)	NA	Very low
Fish oil (1470 mg DHA/147 mg EPA)*	PTSD 0–3 months	1	110	RR: 2.15 (0.20 to 23.04)	NA	Very Low

PTSD, post-traumatic stress disorder, *n*, number of participants included at final assessment, RR, relative risk,

SMD, standard mean difference, CI, confidence interval, NA, not applicable, *only one study for outcome so data not pooled

**Table 4:** Effects of pharmacotherapy for PTSD prevention in child and adolescent participant RCTs.

Pharmacotherapy	Outcome	Comparisons	Participants (*n*)	RR/SMD (95% CI)	*I*^2^	GRADE judgement
Propranolol	PTSD severity 1–3 months	1	20	SMD: 0.01 (−0.87 to 0.89)	NA	Very low
Propranolol	PTSD 1 month–7 years	2	217	RR: 0.48 (0.13 to 1.77)	NA	Very low
Imipramine (vs. chloral hydrate)	ASD severity 0–7 days	1	25	RR: 2.17 (1.04 to 4.51)	NA	Very low

PTSD, post-traumatic stress disorder, ASD, acute stress disorder, *n*, number of participants included at final assessment, RR, relative risk,

SMD, standard mean difference, CI, confidence interval, NA, not applicable

A small positive effect was found for hydrocortisone over placebo on PTSD severity. A larger, but still modest, effect was found for hydrocortisone over placebo on PTSD incidence. The forest plots demonstrating these outcomes are shown in Supplementary Figs. 1 and 2.

## Discussion

This systematic review identified 19 RCTs with 16 included in the meta-analysis and found some evidence for the potential efficacy of hydrocortisone in the prevention of PTSD in adults. There was no evidence to support the efficacy of propranolol in terms of prevention of PTSD or ASD. Considering the paucity of evidence available, it remains difficult to draw firm conclusions on other agents, although no RCT was able to demonstrate an overall beneficial effect without sub-group analysis.

Our results mirror those of previous systematic reviews^24,26^. Sijbrandij et al.^26^ identified 15 studies and evaluated 10 pharmacotherapies while we evaluated 19 and nine respectively. We were unable to evaluate morphine, for which observational studies have associated morphine administration with a decreased PTSD incidence^21^, as there were no RCTs but were able to assess oxytocin, fish oil (1,470 mg DHA/147 mg EPA) and fish oil (224 mg DHA/22.4 mg EPA) due to new evidence. Amos et al. evaluated four hydrocortisone trials, while Sijbrandij et al.^26^ assessed five, and we included six^11,35,36,40,41,43^ (with three in the meta-analysis^11,35,43^). This accumulation of evidence shows continuing interest in evaluating hydrocortisone.

Initiating a therapy within the first six hours post-trauma is thought to be crucial to impeding the disruption to memory consolidation that occurs within this period. Ten studies initiated therapy within 12 h^16,34–37,40,41,43,44,46^—three of propranolol^16,37,46^, six of hydrocortisone^35,36,40,41,43,44^, one of dexamethasone^34^. Five of these hydrocortisone trials^36,40,41,43,44^ initiated therapy within the “golden” six hours, while Delahanty et al.^35^ administered hydrocortisone within a 12-h window and their results suggest that hydrocortisone may still be effective in preventing PTSD outside of a 6-h window. Only one propranolol trial initiated pharmacotherapy within 6 h^16^ and similarly to those initiating therapy later on, also failed to show a preventative effect on PTSD. It remains possible, however, that earlier administration may be more effective in blocking memory consolidation, although the efficacy of hydrocortisone administration at beyond 6 h appears to contradict this, in addition to a limited theoretical basis. It is also likely that memory consolidation is not the only neurobiological process causing PTSD and pharmacological agents may act via different mechanisms or more than one mechanism, oxytocin for example is an anxiolytic in addition to possibly affecting memory consolidation^27^. Disruption of other causative pathways may produce new avenues for pharmacological prevention.

Pragmatically, it is difficult to identify, consent and enrol a participant into a RCT within 6 h of an unexpected trauma, with many studies thus investigating expected trauma (e.g., Intensive Therapy Unit (ITU) admission, cardiac surgery). Hydrocortisone remains a potentially promising intervention for PTSD and is particularly well suited to trauma that necessitates prompt presentation to a hospital setting such as severe injury. Furthermore there is scope for large scale administration in a low resource setting, given its widespread availability as an WHO essential medicine^50^ and its low cost. Given this, it may be better suited to a low resource or disaster setting than a more complicated psychosocial intervention. The possible necessity to administer it within a six-hour time window would hamper its use following many traumatic events and it is only likely to be of pragmatic use if future research confirms it is effective beyond the “golden hours”.

Given the physical conditions of participants and acute hospital settings of the hydrocortisone trials to date, generalisability to other trauma populations is limited and it is possible that co-prescription of other drugs may have subjected the results to confounding. For example, inotropes like noradrenaline are frequently used in septic ITU patients^40^ and correlations have been found between chronic PTSD and raised urinary noradrenaline excretion^51^. It may be that noradrenaline tempers the effect of hydrocortisone and without endogenous noradrenaline administration hydrocortisone may have a greater effect. Furthermore, dosing varied, from 20 mg BD PO hydrocortisone^35^ to a 100 mg IV bolus, followed by a continuous infusion with subsequent tapering^43^. Delahanty et al.’s^35^ work suggested efficacy with an oral formulation, which if validated by subsequent research could increase the usability of hydrocortisone beyond secondary care and into low resource settings.

The analysis of escitalopram, gabapentin, oxytocin, fish oil (1470 mg DHA/147 mg EPA) and fish oil (224 mg DHA/22.4 mg EPA) was limited by the paucity of studies. Only escitalopram was assessed by more than one study. None of these studies administered the treatment within 6 h, and the two escitalopram studies were early treatment, not prevention, studies. It remains possible that earlier administration could be beneficial in preventing PTSD/ASD. Furthermore, the two fish oil studies used vastly different dosages, with neither showing a preventative effect.

Many of the studies examined also investigated concurrent psychiatric co-morbidity. Twelve investigated coexisting depressive symptom severity^22,23,27,34,35,37,38,43–46,48^, but the only significant differences observed between control group and intervention were in those studies investigating hydrocortisone. Hydrocortisone groups reported significantly less depressive symptoms in two studies—Delahanty et al.^35^ and Zohar et al.^44^. Zohar et al.^44^ also found fewer anxiety symptoms in the hydrocortisone group, while Delahanty et al.^35^ found improvements in health related quality of life (HRQL) measures in the intervention group. Likewise, Weis et al.^43^ noted improvements in HRQL. It is possible that hydrocortisone administration may reduce secondary depressive symptoms indirectly by mediating the development of PTSD in traumatised individuals. It is also possible that attenuating the heightened fear response with hydrocortisone may prevent the formation of depression directly, in addition to PTSD. Regardless, both the amelioration of PTSD and depressive symptoms is likely to improve HRQL measures. Future hydrocortisone trials should attempt to measure both depression and PTSD outcomes to further understand this relationship.

There was no apparent difference in effectiveness between interventions administered against placebo and those compared to a different comparator. Only two trials used a non-placebo comparator. Robert et al.^47^ used chloral hydrate when investigating imipramine, while Nishi et al.^39^ used psychoeducation when investigating fish oil (224 mg DHA/22.4 mg EPA). While both of these trials found no significant difference between the two interventions, their design does not allow conclusions to be drawn about actual efficacy as the effect sizes will have been influenced by the comparators’ effects on PTSD outcomes. Two other studies compared an intervention against standard treatment; Rosenberg et al.^48^ found no significant difference between propranolol and standard treatment, while Schelling et al.^41^ found a significant reduction in PTSD symptoms in those treated with hydrocortisone. The magnitude of this result was similar to the other hydrocortisone trials but may have been exaggerated due to the absence of a placebo comparator group.

Many of the RCTs included in our meta-analyses were small and the majority had areas of notable concern for risk of bias; both of these applied to the three RCTs evaluating hydrocortisone. No outcome received a GRADE rating higher than low. This, combined with the lack of studies for many outcomes, limits our confidence in the best current evidence to determine the true effects of early pharmacological intervention to prevent PTSD. This meta-analysis does, however, compile a higher quality of evidence than previously available.

Our work supports previous research investigating the preventative effect of hydrocortisone, but there remains insufficient evidence to recommend its administration routinely. Even in the short term, hydrocortisone use can produce numerous adverse effects, ranging from congestive cardiac failure to insomnia. Of the studies we considered, some reported no side effects^43,44^ while others reported dizziness^35^ and an increased rate of infection/septic shock, hyperglycaemia and hypernatraemia^36,52^. As glucocorticoids have been widely used in medical practice for the past 60 years we have good knowledge of the adverse effects they cause, which may require further medical intervention. While we have sufficient knowledge of the adverse consequences of giving hydrocortisone to people exposed to particular types of trauma, we must further understand the potential benefits and risks in other groups of trauma victims and determine individual factors associated with outcome. Furthermore, severe psychiatric side effects from corticosteroids occur in 6% of patients, with mild to moderate effects occurring in 28%^53^. The most common adverse effects are euphoria and hypomania, although no psychiatric adverse effects were documented in the hydrocortisone RCTs we examined. While side effects are more likely to develop in those requiring higher doses of corticosteroid, dosage does not predict the onset, duration or severity of the adverse reaction^53^. Nonetheless, this suggests caution should be used when prescribing higher dosing regimes. Four RCTs did not exclude patients with a previous psychiatric history, but very limited information was available about the past psychiatric history of their participants. Schelling et al.^40^, however, excluded patients with any psychiatric co-morbidity, and Zohar et al.^44^, excluded patients with a substance use disorder or history of brain trauma. Given the known side effect profile of hydrocortisone, and the reassuring results of studies which did not exclude those with mental illness, we conclude that there is no absolute contra-indication to hydrocortisone for those with pre-existing mental disorder.

Thus, the potential benefit of the medication must be balanced against the potential side effects and patients may be reluctant to receive hydrocortisone in the absence of symptoms. Some of the included studies had problems recruiting participants^35^ and it is probable that a significant proportion of people may not be particularly willing to take medication to prevent PTSD. The reason for reluctance to take medication (with 42.6% refusing SSRI or placebo in one study^42^) is unclear and worthy of further exploration. Reluctance to take medication should not alter evidence-based recommendations to initiate safe and effective pharmacotherapy to prevent PTSD, although is very important with respect to considering implementation and should stimulate future research to consider acceptability in more depth. It seems likely that an optimal approach to offering preventative pharmacotherapy is to target those at highest risk of developing PTSD (for example, those who experienced peritraumatic dissociation) than offering intervention to all trauma-exposed individuals. The evidence examined in this paper suggests that certain agents may be more effective in certain sub-groups of individuals, for example Zohar et al. show the efficacy of escitalopram in victims of intentional trauma, while van Zuiden et al. observe the beneficial effects of oxytocin on trauma victims with high PTSD symptom severity scores. While some of these trials used post-hoc sub-group analyses, this was not the case for all trials, with Delahanty et al.^35^, for example, including only participants with high levels of peritraumatic dissociation. Thus, it is possible that preventative pharmacotherapy will be most efficacious in those at highest risk of developing PTSD, with more severe initial symptom severity.

Similarly when examining dexamethasone, Kok et al.^34^ found a lower prevalence of PTSD and depressive symptoms in women in the intervention group, but were unable to demonstrate lower PTSD/depression rates overall. The trial was the largest included in the meta-analysis, with 2458 participants, and used a stat dose of intravenous dexamethasone (dissimilar to all hydrocortisone trials which used regular administration). The study population was relatively healthy and only admitted to an ITU overnight, leaving them at low risk of developing PTSD compared to other trials and possibly explaining the failure of dexamethasone to prevent PTSD in the population as a whole (but preventing PTSD in women, a higher risk group). Further research should build on this by clarifying which sub-groups best respond to pharmacological prevention.

We conclude that in individuals with no contraindications to its prescription, hydrocortisone could be considered as a preventative intervention for people with severe physical illness or injury, shortly after a traumatic event. While there are general indications that hydrocortisone may be effective, questions remain regarding the sub-groups most likely to benefit and so more research is needed using larger, high quality RCTs to establish the most efficacious use of hydrocortisone in different populations and optimal dosing, dosing window and route. Future research should also clarify other pertinent questions, for instance it is possible that hydrocortisone may impede declarative memory retrieval^54^, which could hinder the recall of testimony required for trauma victims to secure convictions against their perpetrators. Conversely, it is also possible that over-consolidation, or shifts in the degree of contextualisation in consolidated memory in PTSD formation may affect accurate memory recall with similar implications. In addition, further work is required to consider other agents with potential such as oxytocin and opioids, and to develop novel agents informed by our improving neurobiological understanding of PTSD and its development.

## Supplementary information

Supplementary Figure 2

Supplementary Figure 1

PRISMA Checklist

Supplementary Material - Search Strategy

Supplementary Figure 1&2 Legends

Supplementary Tables 1&2
