# Supplementary figures and images for: Pharmacological prevention and early treatment of post-traumatic stress disorder and acute stress disorder: a systematic review and meta-analysis
Source: Transl Psychiatry. 2019 Dec 9;9:334. doi: 10.1038/s41398-019-0673-5 (PMC6901463; doi:10.1038/s41398-019-0673-5)

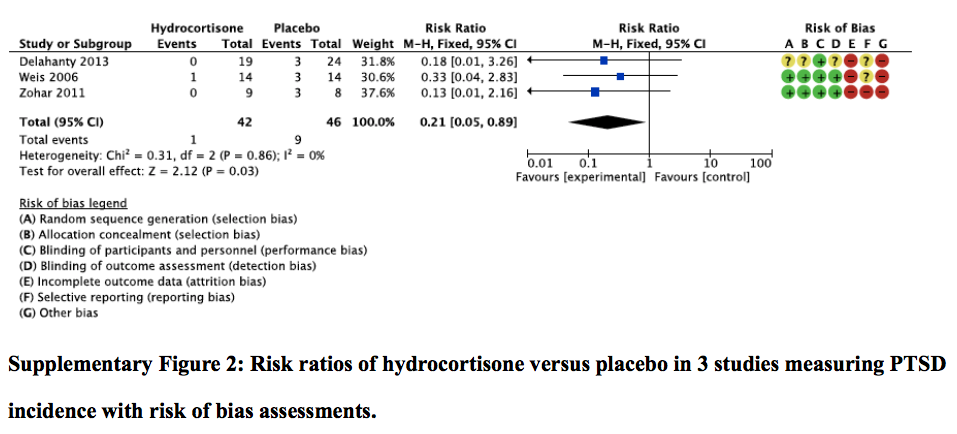

Supplement: Supplementary file 1 — Supplementary Figure 2 [file 41398_2019_673_MOESM1_ESM.png]

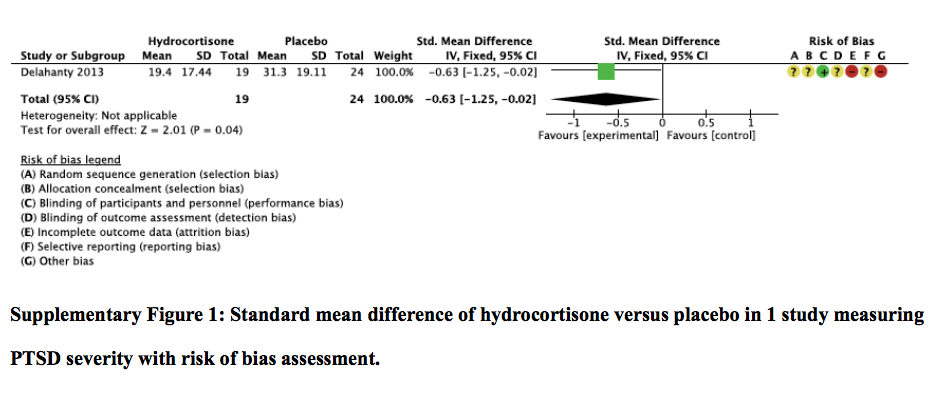

Supplement: Supplementary file 2 — Supplementary Figure 1 [file 41398_2019_673_MOESM2_ESM.png]
