# Supplementary material for: Pharmacological prevention and early treatment of post-traumatic stress disorder and acute stress disorder: a systematic review and meta-analysis
Source: Transl Psychiatry. 2019 Dec 9;9:334. doi: 10.1038/s41398-019-0673-5 (PMC6901463; doi:10.1038/s41398-019-0673-5)
Supplement: Supplementary file 4 — Supplementary Material - Search Strategy [file 41398_2019_673_MOESM4_ESM.docx]

Supplementary Material: Search strategy

Original search carried out on May 6, 2013 with updated search on May 31, 2018.

**Pubmed:**

*MESH terms:*

Stress Disorders, Traumatic [MeSH Terms] AND Prospective studies [MeSH Terms] AND (Adrenergic beta- Antagonists [MeSH Terms] OR Dose-Response Relationship, Drug [MeSH Terms] OR Hydrocortisone/therapeutic use [MeSH Terms] OR Benzodiazepines/pharmacology [MeSH Terms] OR Serotonin Uptake Inhibitors/therapeutic use [MeSH Terms] OR Analgesics, Opioid/therapeutic use [MESH terms]

Stress Disorders, Traumatic [MeSH Terms] AND (Adrenergic beta-Antagonists [MeSH Terms] OR Dose- Response Relationship, Drug [MeSH Terms] OR Hydrocortisone/therapeutic use [MeSH Terms] OR Benzodiazepines/pharmacology [MeSH Terms] OR Serotonin Uptake Inhibitors/therapeutic use [MeSH Terms]) OR Analgesics, Opioid/therapeutic use [MESH terms]

*PsychInfo*

(TX Adrenergic beta-Antagonists OR TX beta-blocker OR TX hydrocortisone OR TX Benzodiazepines OR TX SSRI OR TX Analgesics OR TX opioids OR TX opiates) AND (post-traumatic stress disorders OR PTSD OR posttraumatic Stress)

*Embase*

Keywords: ('trauma' OR 'anxiety' OR 'posttraumatic' OR 'post-traumatic' OR 'PTSD') AND ('propranolol' OR ‘beta-blocker’ OR beta blocker’ OR ‘hydrocortisone’ OR ‘benzodiazepines’ OR ‘SSRI’ OR Analgesics OR opioids OR opiates)

'posttraumatic stress disorder'/exp OR 'psychotrauma'/exp AND 'drug therapy'/exp

Limits: #7 AND ('clinical article'/de OR 'clinical trial'/de OR 'controlled clinical trial'/de OR 'controlled study'/de OR 'drug dose comparison'/de OR 'evidence based medicine'/de OR 'evidence based practice'/de OR 'human'/de OR 'major clinical study'/de OR 'open study'/de OR 'prospective study'/de OR 'randomized controlled trial'/de OR 'randomized controlled trial (topic)'/de OR 'retrospective study’/de)

*Search CCDAN Cochrane Central Register of Controlled Trials:*

Keywords: (propranolol or beta-blocker or hydrocortisone or SSRI or pharmacotherapy OR Analgesics OR opioids OR opiates) and (ptsd or (trauma AND stress) OR 'posttraumatic' OR 'post-traumatic' OR 'PTSD') (Limit; trials)

Additional searches:

Reference lists of earlier reviews and meta-analyses of early interventions or treatments for PTSD. References of the included primary studies.
