# Supplementary material for: Pharmacological prevention and early treatment of post-traumatic stress disorder and acute stress disorder: a systematic review and meta-analysis
Source: Transl Psychiatry. 2019 Dec 9;9:334. doi: 10.1038/s41398-019-0673-5 (PMC6901463; doi:10.1038/s41398-019-0673-5)
Supplement: Supplementary file 5 — Supplementary Figure 1&2 Legends [file 41398_2019_673_MOESM5_ESM.docx]

Supplementary Figure Legends

Supplementary Figure 1: Standardised mean difference of hydrocortisone versus placebo in 1 study measuring PTSD severity with risk of bias assessment.

Supplementary Figure 2: Risk ratios of hydrocortisone versus placebo in 3 studies measuring PTSD incidence with risk of bias assessments.
