# Supplementary material for: Pharmacological prevention and early treatment of post-traumatic stress disorder and acute stress disorder: a systematic review and meta-analysis
Source: Transl Psychiatry. 2019 Dec 9;9:334. doi: 10.1038/s41398-019-0673-5 (PMC6901463; doi:10.1038/s41398-019-0673-5)
Supplement: Supplementary file 6 — Supplementary Tables 1&2 [file 41398_2019_673_MOESM6_ESM.docx]

Supplementary Table 1 & 2

|  | **Risk of bias criteria** | | | | | | | |
| --- | --- | --- | --- | --- | --- | --- | --- | --- |
|  | **Random sequence generation** | **Allocation concealment** | **Blinding of participants and personnel** | **Blinding of outcome assessment** | **Incomplete outcome data** | **Selective reporting** | **Other sources of bias** | **Total no of low risk domains** |
| Delahanty et al | Unclear | Unclear | Low Risk | Unclear | High Risk | Unclear | High Risk | 1 |
| Denke et al | Low Risk | Low Risk | Low Risk | Unclear | High Risk | Unclear | High Risk | 3 |
| Hoge et al | Unclear | Unclear | Unclear | Unclear | High Risk | Unclear | High Risk | 0 |
| Kok et al | Low Risk | Low Risk | Low Risk | Low Risk | Low Risk | Unclear | High Risk | 5 |
| Matsuoka et al | Low Risk | Low Risk | Unclear | Low Risk | Low Risk | Low Risk | Low Risk | 6 |
| Nishi et al | Low Risk | Low Risk | High Risk | Low Risk | Low Risk | Low Risk | Low Risk | 6 |
| Pitman et al | Unclear | Low Risk | Low Risk | Unclear | High Risk | High Risk | High Risk | 2 |
| Schelling et al 2001 | Unclear | Low Risk | Low Risk | Low Risk | High Risk | Unclear | High Risk | 3 |
| Schelling et al 2004 | Unclear | High Risk | Unclear | High Risk | High Risk | Unclear | High Risk | 0 |
| Shalev et al | Low Risk | Low Risk | Low Risk | Low Risk | High Risk | Low Risk | High Risk | 5 |
| Stein et al | Low Risk | Low Risk | Low Risk | Low Risk | High Risk | Low Risk | High Risk | 5 |
| Suliman et al | Low Risk | Low Risk | Low Risk | Low Risk | High Risk | Low Risk | High Risk | 5 |
| Van Zuiden et al | Low Risk | Low Risk | Low Risk | Low Risk | Low Risk | High Risk | High Risk | 4 |
| Weis et al | Low Risk | Low Risk | Low Risk | Low Risk | High Risk | Unclear | High Risk | 4 |
| Zohar et al 2011 | Low Risk | Low Risk | Low Risk | Low Risk | High Risk | High Risk | High Risk | 4 |
| Zohar et al 2018 | Unclear | Low Risk | Low Risk | Unclear | High Risk | Unclear | Low Risk | 3 |
| **Supplementary Table 1: Risk of bias assessments for adult studies** | | | | | | | | |

|  | **Risk of bias criteria** | | | | | | | |
| --- | --- | --- | --- | --- | --- | --- | --- | --- |
|  | **Random sequence generation** | **Allocation concealment** | **Blinding of participants and personnel** | **Blinding of outcome assessment** | **Incomplete outcome data** | **Selective reporting** | **Other sources of bias** | **Total no of low risk domains** |
| Nugent | Unclear | Low Risk | Low Risk | Low Risk | High Risk | Unclear | High Risk | 3 |
| Robert et al | High Risk | Low Risk | Low Risk | Unclear | High Risk | High Risk | High Risk | 2 |
| Rosenberg et al | Unclear | Unclear | Unclear | Low Risk | High Risk | Unclear | High Risk | 1 |
| **Supplementary Table 2: Risk of bias assessments for child and adolescent studies** | | | | | | | | |
